# Dengue Virus Induced COX-2 Signaling Is Regulated Through Nutrient Sensor GCN2

**DOI:** 10.3389/fimmu.2020.01831

**Published:** 2020-08-13

**Authors:** Sumbul Afroz, Srikanth Battu, Jeevan Giddaluru, Nooruddin Khan

**Affiliations:** ^1^Department of Biotechnology and Bioinformatics, School of Life-Sciences, University of Hyderabad, Hyderabad, India; ^2^Laboratory of Molecular Cell Biology, Centre for DNA Fingerprinting and Diagnostics (CDFD), Hyderabad, India; ^3^Department of Animal Biology, School of Life-Sciences, University of Hyderabad, Hyderabad, India

**Keywords:** dengue virus, nutrient sensor GCN2, COX-2/ PGE_2_ signaling, transcriptional regulation, NF-κB pathway

## Abstract

Nutrient sensor GCN2 plays a crucial role in the maintenance of cellular homeostasis during the condition of amino acid deprivation. Dysfunction in the GCN2 signaling underlies several chronic metabolic diseases. Recent studies highlight the anti-viral potential of GCN2 against RNA viruses such as Sindbis and HIV. However, its effect on dengue virus (DENV) pathogenesis remains poorly understood. Herein, we report that GCN2 deficient cells show increased DENV replication and viral yield in the culture supernatants compared to WT cells infected with DENV. Notably, enhanced DENV replication in GCN2^−/−^ cells is associated with increased COX-2/PGE_2_ signaling. Conversely, GCN2 overexpression/activation effectively contains DENV infection by inhibiting COX-2/PGE_2_ signaling. Mechanistically, deletion of GCN2 triggers enhanced production of COX-2/PGE_2_ through profound activation of Iκκ-NF-κB signaling pathway. Altogether our results unveil a hitherto unrecognized role of GCN2 in DENV pathogenesis, thereby suggesting that targeting the GCN2 pathway might offer a novel therapeutic intervention against DENV infection.

## Introduction

Eukaryotic cells orchestrate innate defense mechanism of translational arrest in response to wide variety of cellular stresses, including heat shock, starvation, and viral infection (1). Four different intracellular kinases that are activated by a particular stress stimulus exist in eukaryotes such as general control nonderepressible 2 kinase (GCN2), activated during amino acid or serum starvation and UV stress (2), double stranded RNA dependent protein kinase (PKR), senses double stranded RNA (3), heme-regulated inhibitor (HRI), activated during heme deficiency and heat shock (4), while PKR-like endoplasmic reticulum kinase (PERK) is activated by unfolded protein response (5). Upon activation, each of these kinases phosphorylate the alpha subunit of eukaryotic translational initiation factor (eIF2α), leading to the decreased eIF2-GTP-Met-tRNAi^Met^ complex resulting in translational arrest (6). Though PKR is well-known for defense against variety of viral infections (7), the redundancy of PKR against various viruses exist (8), indicating the presence of other antiviral pathways. Recent studies have highlighted that GCN2 shows potent anti-viral responses against Sindbis virus (9) and human immunodeficiency virus (HIV) (10). However, the implication of GCN2 pathway in Dengue pathogenesis has been poorly defined.

With an infection rate of around 400 million annually, Dengue virus (DENV) infection embodies a global threat (11, 12). Several compelling reports reveal that exacerbated inflammation during DENV infection accounts for the pathogenesis of severe dengue disease. This is predominantly attributed to the activation of cyclooxygenase/postraglandin-E_2_ (COX-2/PGE_2_) signaling pathways, which eventually leads to a burst of pro-inflammatory chemokine production including CCL21 and MIP-3β (13). Concomitantly COX-2/PGE_2_ have also been shown to favor DENV replication and pathogenesis (14). Given the significance of COX-2/PGE_2_ signaling pathway during DENV infection it becomes imperative to understand the mechanism through which DENV activates this pathway in the innate immune cells for its survival and evasion of host anti-viral responses. Recently, we have shown that nutrient sensor GCN2, plays a crucial role in controlling intestinal inflammation by restricting the production of IL-1β (15). In addition it has been shown that activation of GCN2 triggers antiviral effect against RNA viruses including SV, VSV (9), and HIV (16). This tempted us to dissect the mechanistic insights through which GCN2 mounts antiviral responses including regulation of inflammatory responses during DENV infection.

Herein, using DENV infection model, we show that GCN2 activation negatively regulates the potent inflammatory COX-2/PGE_2_ pathway by interfering with Iκκ-NF-κB signaling. Deletion of GCN2 resulted in hyperactivation of COX-2/ PGE_2_, while its over-expression had an opposite effect during DENV infection. Further, we demonstrate that GCN2 deficient cells were highly susceptible to infection with all the DENV serotypes as well as show higher accumulation of DENV dsRNA intermediate, indicating that GCN2 has an anti-viral effect against DENV by impeding DENV replication. Overall, our results identify GCN2 as a crucial component of the host's defense system, thereby suggesting that GCN2 could be a potential therapeutic target towards design of novel anti-viral strategies against DENV infection.

## Materials and Methods

### Cells, Chemicals, and Plasmids

Liver human hepatoblastoma cells (HepG2), and Vero cells were cultured in complete DMEM (Gibco) media consisting of 10% FBS (GIBCO), 100 units/ml Penicillin, 1 μg/ml Streptomycin, L-Glutamine, and 1 mM sodium pyruvate. DR-Wildtype (ATCC CRL2977) and GCN2-KO-DR (ATCC CRL2978) MEFs were purchased from American Type Culture Collection (ATCC) and cultured in complete DMEM growth medium as described earlier (15). Halofuginone hydrobromide (trans-[±]-7-Bromo-6-chloro-3-[3-(3-hydroxy-2-piperidinyl)-2-oxopropyl]-4[3H]) quinazolinonemonohydrobromide and Actinomycin-D were purchased from Sigma-Aldrich; Lipofectamine 2000 (Invitrogen, Life Technologies). pNifty-Luc was purchased from Invivogen and pKM2L-phCOX-2 plasmid was purchased from RIKEN Bioresource Center (DNA Bank), Japan. pCDNA3.1-Flag-GCN2 plasmid was a kind gift from Prof. David Ron's lab, Cambridge University.

### Viruses

Dengue virus serotype 1 (Hawaii), Dengue virus serotype 2 (TR1751), and Dengue virus serotype 4 (Columbia 1982) were used in this study and propagated in C636 cell line as described earlier (17, 18). Briefly 90% confluent layers of C636 cells were infected at a multiplicity of infection (moi) of 0.01 and incubated for 7 days. Post incubation, the supernatant was collected, clarified by centrifugation and stored at −80°C.The titer of the viruses was evaluated by Focus forming assay (FFU) on Vero cells.

### Isolation of Primary Human Monocytes

Human monocytes were isolated from blood collected from healthy volunteers as described earlier through double gradient centrifugation method (19). Briefly, peripheral blood nuclear cells (PBMCs) were isolated after layering the blood on Ficoll solution (1.077 g/ml) (Histopaque, Sigma Aldrich) followed by centrifugation at 400 × g for 30 min at 25°C. The white buffy coat ring containing the PBMCs was separated which was further layered on iso-osmotic Percoll solution (1.131 g/ml) for the second density gradient for the separation of monocytes from lymphocytes.

### Immunoblotting

Mock or DENV infected cells were washed with chilled 1x PBS, lysed using mammalian lysis buffer supplemented with 1X Protease inhibitor cocktail (Sigma Aldrich) as described earlier (15). Total proteins in the lysates were estimated using Bicinchoninic Acid (BCA) protein assay (G Biosciences) and equal concentration of proteins was loaded on Polyacrylamide SDS-PAGE (10–12% Tricine gel). Proteins were transferred on to a nitrocellulose membrane (Pall Corporation) through electroblotting. Further, membranes were blocked with 5% skimmed milk (HiMedia) or 5% BSA (for phospho- blots; Sigma Aldrich) in 1X PBS and incubated with primary antibodies (Abs) at 4°C overnight. The primary antibodies used were rabbit Dengue Virus NS3 (GeneTex), goat anti-COX2 (Santa Cruz), mouse anti-p65 (Santa Cruz), mouse anti-p50 (Santa Cruz), rabbit anti phospho NF-κBp65, rabbit anti-NF-κBp65 (total), rabbit anti-phospho IKKα/β, rabbit anti-IKKα, mouse anti-IKKβ, rabbit anti phospho IκBα, rabbit anti IκBα (Cell Signaling Technology). Following primary anti body incubation, membranes were washed with 1X TBST and incubated with appropriate secondary Abs for 1 h at room temperature. Proteins bands were visualized using femtoLUCENT™ chemiluminescent substrate (G Biosciences) and chemiluminiscence was captured by ChemiDoc Touch Imaging System (BioRad). Densitometry analysis of protein bands on blots was done using NIH software Image J.

### PGE_2_ Assay

PGE_2_ was estimated in the culture supernatants of DENV infected HepG2 cells or WT and GCN2^−/−^ MEFs using PGE_2_ Express ELISA kit (Cayman Chemical) according to manufacturer's instructions. Briefly, HepG2 cells or WT and GCN2^−/−^ MEFs were infected with DENV for various time-points and the supernatant was collected for PGE_2_ estimation. Anti-mouse IgG coated plates were incubated with culture supernatants along with PGE_2_ Tracer and PGE_2_ Express monoclonal Ab at room temperature for 60 min. Following five washes with Wash Buffer, the plate was developed using freshly prepared Ellman's Reagent. Absorbance was measured at a wavelength between 405 and 420 nm using Tecan Microplate reader.

### Immunofluorescence

WT or GCN2^−/−^ MEFs were infected with DENV at moi 3 and incubated for indicated time points (as indicated in the results section). Post incubation, the infected cells were fixed with 4% paraformaldehyde in 1X PBS for 15 min followed by permeabilization with 0.2% Triton-X 100 for 20 min at room temperature. The cells were further blocked with 10% FBS or 1% BSA for 1 h at room temperature. Following washes with 1X PBS, cells were incubated with primary Ab for 2 h at 37°C followed by incubation with fluorescent secondary Abs, Alexa Fluor-488 (Invitrogen) at 37°C for 1 h. Next, cells were washed three times with 1X PBS and immunostained cells were finally mounted using Prolonged Gold Anti-fade Reagent containing DAPI (Invitrogen) or Vectashield Mounting Medium with DAPI (Vector Laboratories). The primary antibodies used were goat anti-COX2 (Santa Cruz), mouse anti-Dengue 1, 2, 3, 4 (GeneTex), mouse anti dsRNA-J2 (English and Scientific Consulting Kft.), and mouse anti-p65 (Santa Cruz). The immunofluorescence images were captured under 63X oil based objective using a LSM510 confocal microscope (Zeiss). Further, the images were processed and analyzed in Zeiss LSM5 image browser. The analysis of intensity profiles was carried out using Fiji/Image J software (NIH).

### Transient Transfections

*In-vitro* transient transfections were carried out in HepG2 cells using Lipofectamine 2000 reagent according to manufacturer instructions. Briefly, the cells were transfected at a confluency of 70–80% in reduced serum media, OptiMEM (Gibco) with either pCDNA3-Flag-GCN2 alone or in-combination with pKM2L-phCOX-2, pNifty-Luc for 6 h, following which the media was replaced with complete DMEM and incubated for 48 h, after which functional analysis was done.

### qRT-PCR

Total RNA from DENV infected cells under different experimental conditions was isolated using TRIZOL (Invitrogen). One−2 μg of purified RNA was reverse transcribed into cDNA using Verso cDNA synthesis kit (Thermo scientific) according to manufacturer's instructions. qRT-PCR was performed using Master cycler ep realplex (Eppendorf) or QuantStudio 5 (Applied Biosystems). Thirty−50 ng cDNA was amplified using gene specific primers (human COX-2: *FP: 5*′*CCTTCCTCCTGTGCCTGATG 3*′*RP: 5*′*ACAATCTCATTTGAATCAGGAAGCT 3*′, human GAPDH *FP: GGAGCGAGATCCCTCCAAAAT RP: GGCTGTTGTCATACTTCTCATGG*, mouse COX-2: *FP: 5*′*CCATGTCAAAACCGTGGTGAATG 3*′*RP: 5*′*ATGGGAGTTGGGCAGTCATAG 3*′, mouse GAPDH *FP: 5*′*AAGGTCATCCCAGAGCTGAA 3*′*RP: 5*′*CTGCTTCACCACCTTCTTGA 3*′*,)*. The relative mRNA expression of each gene was normalized to house-keeping gene GAPDH.

### Subcellular Fractionation

Nuclear and Cytoplasmic fractions were prepared as described previously (20) with slight modifications. Briefly, the cell pellets were resuspended gently in ~100 μl (five times pellet volume) cold Cytoplasmic Extract (CE) Buffer (10 mM HEPES, 1 mM EDTA, 0.075% (v/v) NP40, 60 mM KCl, 1 mM DTT and 1 mM PMSF, pH 7.6) and incubated on ice for 10 min. The homogenate was centrifuged at 1,500 rpm for 5 min and the cytoplasmic extract was collected in a fresh tube. The pellet containing the nuclei was washed with CE buffer without NP40 by centrifugation at 1,500 rpm for 4 min. Approximately, 40 μl (one pellet volume) Nuclear extract (NE) buffer (20 mM Tris Cl, 420 mM NaCl, 1.5 mM MgCl_2_, 0.2 mM EDTA, 1 mM PMSF, and 25% (v/v) glycerol, adjusted to pH 8.0) was added to the pellet and incubated on ice for 15 min with intermittent vortexing. Both the CE and NE were centrifuged at maximum speed at 4°C to pellet any nuclei. The contents of CE and NE tubes were transferred to separate clean tubes and run on SDS-PAGE followed by immunoblotting.

### Luciferase Activity Assay

To evaluate the effect of GCN2 on COX-2 promoter activity, HepG2 cells were transfected with a COX-2 promoter-reporter plasmid pKM2L-phCOX-2-Renilla-Luc alone or in-combination with pcDNA3.1-Flag-GCN2 or vector control and incubated for 48 h. To evaluate the potential of GCN2 to counteract the activation of NF-κB pathway, we transfected HepG2 cells with a NF-κB responsive luciferase plasmid pNifty-Luc alone or with pcDNA3.1-Flag-GCN2 for 48 h. Following incubation, luciferase activity assay was performed using Dual-Glo® Luciferase Assay Kit (Promega) according to manufacturer's instructions. Briefly, Dual-Glo® Luciferase Reagent was added to the transfected cells for lysis following which the firefly luminescence was measured using Tecan microplate reader. For the estimation of Renilla luminescence, Dual-Glo® Stop & Glo® Reagent was further added to the cell lysates after which the luminescence reading was taken in a Tecan microplate reader.

### Quantification of Viral Yields

Infectious Dengue virus yield was quantified in the culture supernatants through FACS and was represented in the form of FACS infectious unit/ml (FACSIU/ml) as described earlier (21). Vero cells seeded overnight at a density of 5 × 10^4^ cells in a 12-well plate, were incubated with 350 μl of culture supernatants of DENV-2 infected WT and GCN2^−/−^ cells or DENV-2 infected cells overexpressing GCN2 or containing empty vector for 90 min at 37°C with frequent shaking at 15 min interval. Post- incubation the culture supernatant was removed and replaced with fresh DMEM medium containing 2%FBS and further incubated for 24 h. After 24 h the cells were washed, scarped and fixed using 4% paraformaldehyde. Fixed cells were permeabilized using 1X Intracellular Staining Perm Wash Buffer (Bio Legend) followed by incubation with anti-Flavivirus [D1-4G2-4-15(4G2)] antibody diluted in 1X Perm Wash or respective isotype control at 4°C overnight to detect Dengue virus. Following washes, the cells were incubated with Alexa-488-IgG (Invitrogen) for 1 h at 37°C, washed, resuspended in FACS buffer and acquired on BD LSR Fortessa™. The FACS data was analyzed using FlowJo software. The viral titer was determined using the formula as mentioned earlier (21): FACS infectious units (IU)/ml = [average percent of positive DENV infected cells – average percent of positive mock infected cells) × (total number of cells in well) × (dilution factor)] / (volume of inoculum added to cells).

### Focus Forming Unit Reduction Assay (FFURA)

The anti-viral potency of GCN2 was evaluated in terms of reduction in the number of infectious DENV virions in GCN2 overexpressed Vero cells through Focus Forming Reduction Assay as described earlier (17). Vero cells were transfected with pcDNA3.1-Flag-GCN2 expressing constructed and incubated for 36 h, following which the transfected cells were infected with DENV-2 (moi 2). Post viral adsorption, the cells were overlaid with 1.5% carboxymethyl cellulose supplemented conditioned media and incubated for 4 days. Post incubation, the overlay media was removed, the cells were washed with 1X PBS, fixed with 4% paraformaldehyde, permeabilized with 0.2% Triton X-100 and stained with an anti-dengue monoclonal antibody (GeneTex, USA) overnight at 4°C followed by incubation with HRP-linked anti-mouse secondary Ab for 1 h at 37°C. The viral foci was observed after developing with DAB substrate. The percentage of Foci reduction was compared with only vector transfected controls and calculated using the formula mentioned earlier (17).

### Statistical Analysis

The statistical analysis were performed using Graph Pad Prism 7.0 software. The significance between data was assessed through Student's *t*-test, one-way analysis of variance (ANOVA) or 2-way ANOVA as indicated in each of the figure legends. Data with *P* < 0.05 was considered significant.

## Results

### GCN2 Exhibits Antiviral Effect Against Dengue Virus

**Figure 1:**
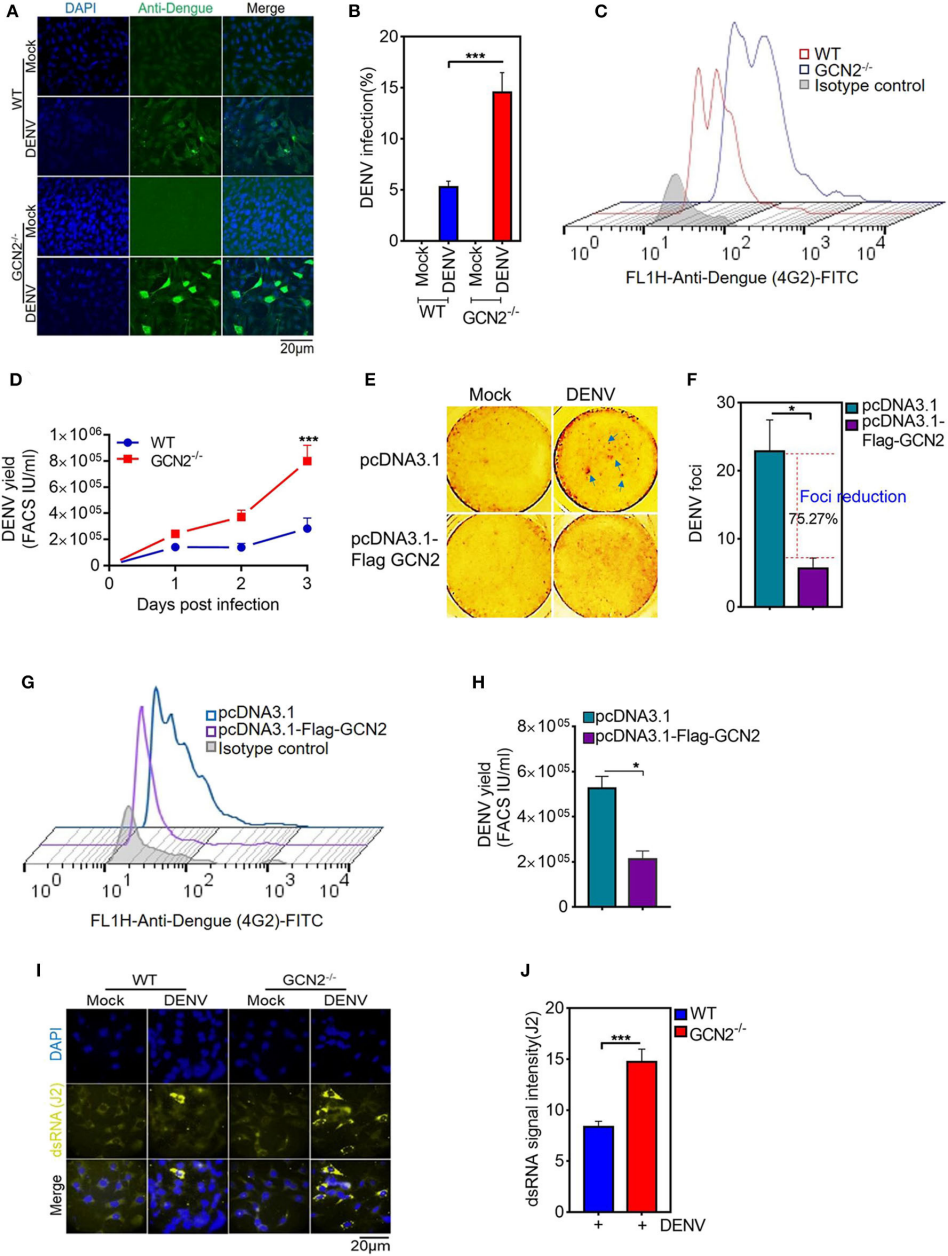
GCN2 restrains DENV infection by interfering with its replication. WT and GCN2^−/−^ MEFs were mock infected or infected with DENV-2 (moi 3) for 36 h for analysis of DENV pathogenesis. The cells were immunostained with Anti-Dengue (green) antibody to stain DENV infected cells. **(A)** Confocal microscopy image showing the no. of DENV-2 positive cells; **(B)** Quantification of percentage of infection in WT and GCN2^−/−^ MEFs using Image J (NIH) software. Data is represented as mean ± SEM from 10 different fields of three independent experiments. **(C,D)** WT and GCN2^−/−^ MEFs were mock infected or infected with DENV-2 (moi 2) for up to 3 days and the culture supernatants were collected. Vero cells were infected with the culture supernatants for 24 h and the virus yields in the culture supernatants was quantified through FACS. **(C)** Representative FACS histogram plot showing DENV infection. **(D)** Graph showing the viral titer represented as FACS IU/ml in the culture supernatant of DENV infected WT and GCN2^−/−^ MEFs at the indicated time points. Data is mean ± SEM from three independent experiments. **(E,F)** Vero cells were transfected with pcDNA3.1-Flag-GCN2 or vector (pcDNA3.1) control followed by DENV-2 infection at a moi 2 and further overlaid with 1.5% carboxymethylcellulose and incubated for 4 days. **(E)** Foci forming unit reduction assay (FFURA) was performed using DENV foci immunostaining method. **(F)** Graph (fold over uninfected mock controls) represents percentage of Foci reduction in GCN2 overexpressed cells. **(G,H)** Vero cells were transfected with pcDNA3.1-Flag-GCN2 or vector (pcDNA3.1) control followed by DENV-2 (moi 3) infection for 48 h. The culture supernatant was collected and infectious viral yield was quantified through FACS by infecting Vero cells for 24 h with the culture supernatant. **(G)** Representative FACS histogram plot showing DENV infection. **(H)** Graph showing the viral titer represented as FACS IU/ml in the culture supernatant of DENV infected cells overexpressing GCN2. Data is mean ± SEM from three independent experiments. **(I)** Analysis of dsRNA accumulation in DENV-2 (moi 3) infected WT and GCN2^−/−^ MEFs at 36 hpi by immunofluorescence. J2 antibody was used to detect DENV dsRNA intermediate. Alexa-Fluor 488 conjugated secondary antibodies was used to detect primary antibody, respectively. **(J)** Quantification of dsRNA (J2 signal intensity) from **(I)** using Image J software. J2 signal was normalized to the background. Data is mean ± SEM of three independent experiments. *P < 0.05, ***P < 0.001 was considered significant. Statistical analysis was done using two-tailed unpaired Student's *t-*test.

Accumulating evidence highlights a key role for GCN2 in triggering anti-viral responses against RNA viruses including SV, VSV (9), and HIV (16). To assess the impact of GCN2 on DENV infectivity, we first infected WT or GCN2^−/−^ MEFs with DENV-2 virus for 48 h at a moi of 3 and visualized the overall percentage of DENV infected cells by confocal microscopy. We found that there was greater viral infection in GCN2^−/−^ MEFs as compared to WT MEFs (Figures 1A,B). Next, we determined the viral yields in WT and GCN2^−/−^ MEFs 3 days post DENV-2 infection. The viral yields were significantly increased by ~4-fold in GCN2^−/−^ cells as compared to WT cells (Figures 1C,D). As occurred with DENV-2, DENV-1 and 4 infection was also largely enhanced in GCN2^−/−^ MEFs as compared to WT MEFs (Figures S1A,B) confirming that cells lacking GCN2 are more susceptible to DENV infection. Next, we examined the anti-DENV activity of GCN2 on DENV foci formation in GCN2 overexpressing Vero cells. Vero cells were transiently transfected with either vector alone or GCN2 overexpressing plasmid for 36 h followed by DENV-2 (moi 5) infection for further 24 h. DENV-2 foci formation was abrogated in cells transfected with GCN2 as compared to vector transfected cells implying that GCN2 overexpression perturbs DENV infection in Vero cells (Figures 1E,F). The infectious viral yields in GCN2 overexpressed cells were also significantly reduced as observed by ~3-fold reduction in viral titres in the culture supernatant of cells overexpressing GCN2 as compared to empty vector post DENV-2 infection (Figures 1G,H). To examine whether GCN2 dampens DENV virion formation by interfering with its replication, we assessed the intracellular accumulation of DENV double stranded (dsRNA) intermediate through confocal microscopy using J2 antibody, (22) which specifically detects dsRNA but not tRNA or cellular rRNA, and has been previously used to detect Flavivirus replication complexes (23, 24). We infected WT and GCN2^−/−^ MEFs with different DENV serotypes at a moi of 3 followed by incubation for 36 h. DENV infected GCN2^−/−^ MEFs exhibited greater dsRNA staining in the cytoplasm suggesting greater accumulation of dsRNA in the cytoplasm of GCN2^−/−^ MEFs as compared to WT MEFs upon DENV-2 (Figures 1I,J) infection as well as other DENV serotypes (DENV-1 and DENV-4) (Figures S1C,D). Altogether, these results suggest an inhibitory effect of the GCN2 pathway on DENV replication and infection.

### GCN2 Suppresses DENV Infection by Impeding DENV-Induced COX-2/PGE_2_ Signaling

Growing evidences suggest that GCN2 plays a crucial role in controlling inflammation, however, its role in DENV induced inflammation is still not clear. We examined whether GCN2 activation affects the levels of COX-2/PGE_2_, one of the key inflammatory mediators associated with severe dengue. We first determined the levels of COX-2 in HepG2 cells infected with DENV. We observed a substantial increase in COX-2 expression (Figure S2A) as well as COX-2 activity estimated as production of PGE_2_ as DENV-2 infection advances (Figure S2B). Similarly, we observed increase in COX-2 expression in DENV infected primary human monocytes as the infection time progresses (Figure S2C). These data are in line with previous studies which have shown up-regulation in the COX-2 levels upon DENV infection (13, 14).

**Figure 2:**
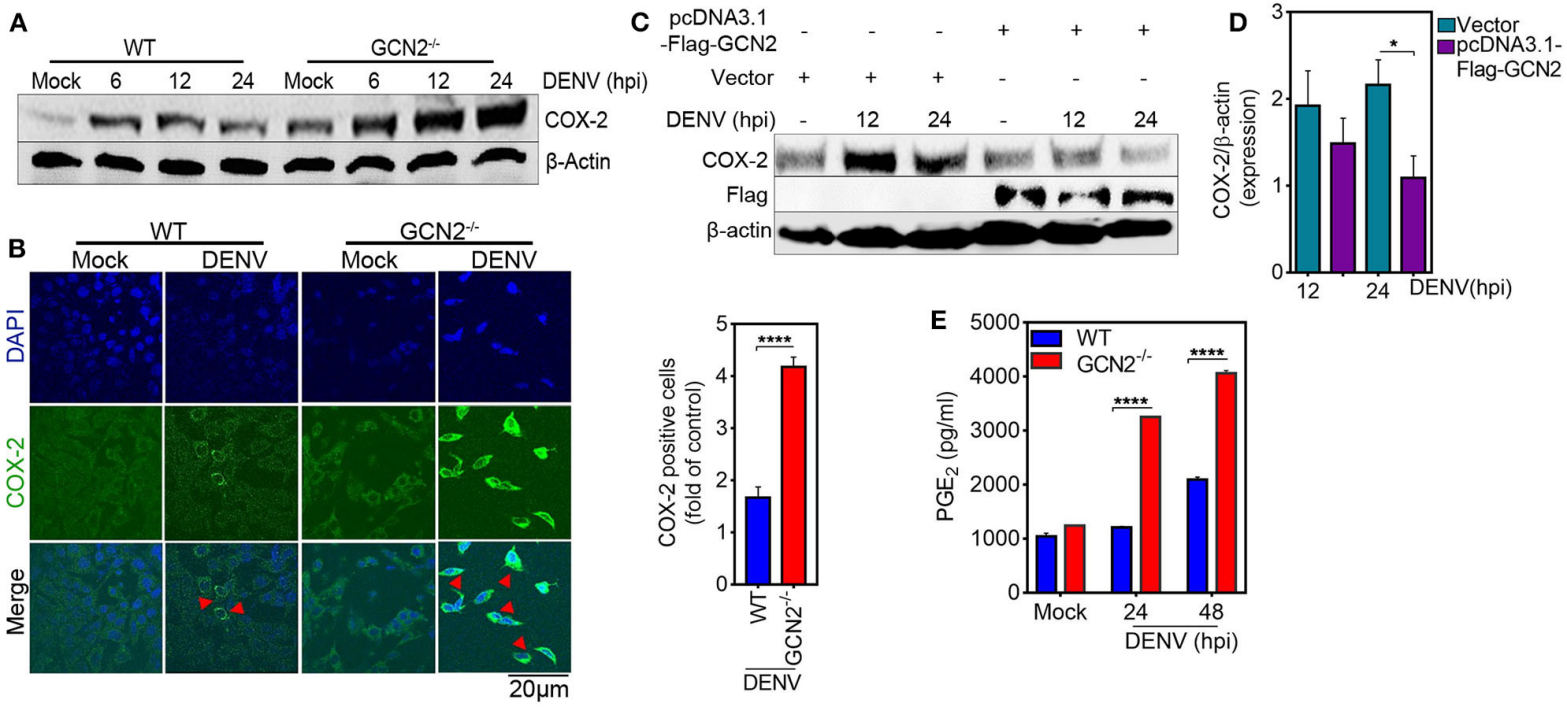
GCN2 suppresses DENV induced COX-2 signaling. **(A)** Immunoblot representing the COX-2 expression in DENV-2 (moi 3) infected WT or GCN2^−/−^ MEFs at 6, 12, and 24 hpi. **(B)** Confocal microscopy imaging of COX-2 expression in DENV-2 (moi 3) infected WT or GCN2^−/−^ MEFs 24 h post infection (left). Bar graph showing the quantification of no. of COX-2 positive cells in DENV-2 infected WT or GCN2^−/−^ MEFs (right). The cells were counted in at-least 10 different fields using Image J (NIH) software; data shown is the mean ± SEM of three independent experiments. **(C,D)** HepG2 cells were transfected with pcDNA3.1-Flag-GCN2 plasmid or vector control followed by DENV-2 (moi 3) infection for the indicated time points. **(C)** COX-2 expression level was assessed through western blotting. **(D)** Densitometry analysis of COX-2 protein relative to β-actin was done using Image J software (NIH) and represented as bar graph. Graph represents data as mean ± SEM from three independent experiments. **(E)** Graph representing the PGE_2_ levels in the culture supernatants of DENV-2 (moi 3) infected WT or GCN2^−/−^ MEFs at indicated time-points; data shown is mean ± SEM of at least two independent experiments performed in triplicates. *P < 0.05 and ****P < 0.0001 was considered significant. Statistical significance was done using two-tailed unpaired Student's t-test in **(B)** and **(D)** and 2-way Anova in **(E)**.

To further elucidate the role of GCN2 in DENV induced COX-2 production, we first examined the levels COX-2 expression in the GCN2 depleted cells infected with DENV-2. Immunoblot analysis showed a profound increase in COX-2 expression in GCN2^−/−^ mouse embryonic fibroblasts (MEFs) as compared to wild type (WT) MEFs upon 6, 12, and 24 h post DENV-2 infection (Figure 2A), which was further confirmed by confocal microscopy analysis with GCN2^−/−^ MEFs showing greater proportion of COX-2 positive cells coincident with DENV-2 infection (Figure 2B). Conversely, we over expressed GCN2 in HepG2 cells and evaluated the expression pattern of COX-2. Our results showed that COX-2 expression was suppressed in GCN2 overexpressed HepG2 cells as compared to vector control during DENV-2 infection at later time points (Figures 2C,D). These data suggest an inverse correlation between the GCN2 signaling and COX-2/PGE_2_ pathways during DENV infection. COX-2 metabolizes arachidonic acid from the cell membrane and converts it into PGE_2_, PGD_2_, PGI_2_, PGF_2α_, and thromboxane A_2_ through the generation of the intermediate PGH_2_ (25). Through the production of PGE_2_, COX-2 has been shown to contribute to the clinical manifestations of inflammation during DENV infection (13). We therefore evaluated the impact of GCN2 on PGE_2_ production, an estimate of COX-2 activity. We observed high levels of PGE_2_ in the culture supernatant of DENV-2 infected GCN2^−/−^ MEFs as compared to WT MEFs as the infection progresses (Figure 2E). Collectively, these results suggest that GCN2 may antagonize inflammatory responses during DENV infection by interfering with COX-2/PGE_2_ signaling.

To determine whether GCN2 mediated control of DENV pathogenesis is dependent on COX-2, we pharmacologically inhibited COX-2 using a COX-2 specific inhibitor, Celecoxib (26, 27), in WT and GCN2^−/−^ cells and assessed the levels of DENV. We observed significant reduction in DENV infection both in WT and GCN2^−/−^ cells treated with celecoxib (Figures S3A,B), suggesting that increased DENV replication in GCN2^−/−^ cells is due to heightened production of COX-2. Altogether, these results suggest that GCN2 orchestrates anti-viral responses against DENV by directly suppressing its replication and simultaneously limiting COX-2 production.

### GCN2 Dysregulates COX-2 Expression at the Level of Transcription

**Figure 3:**
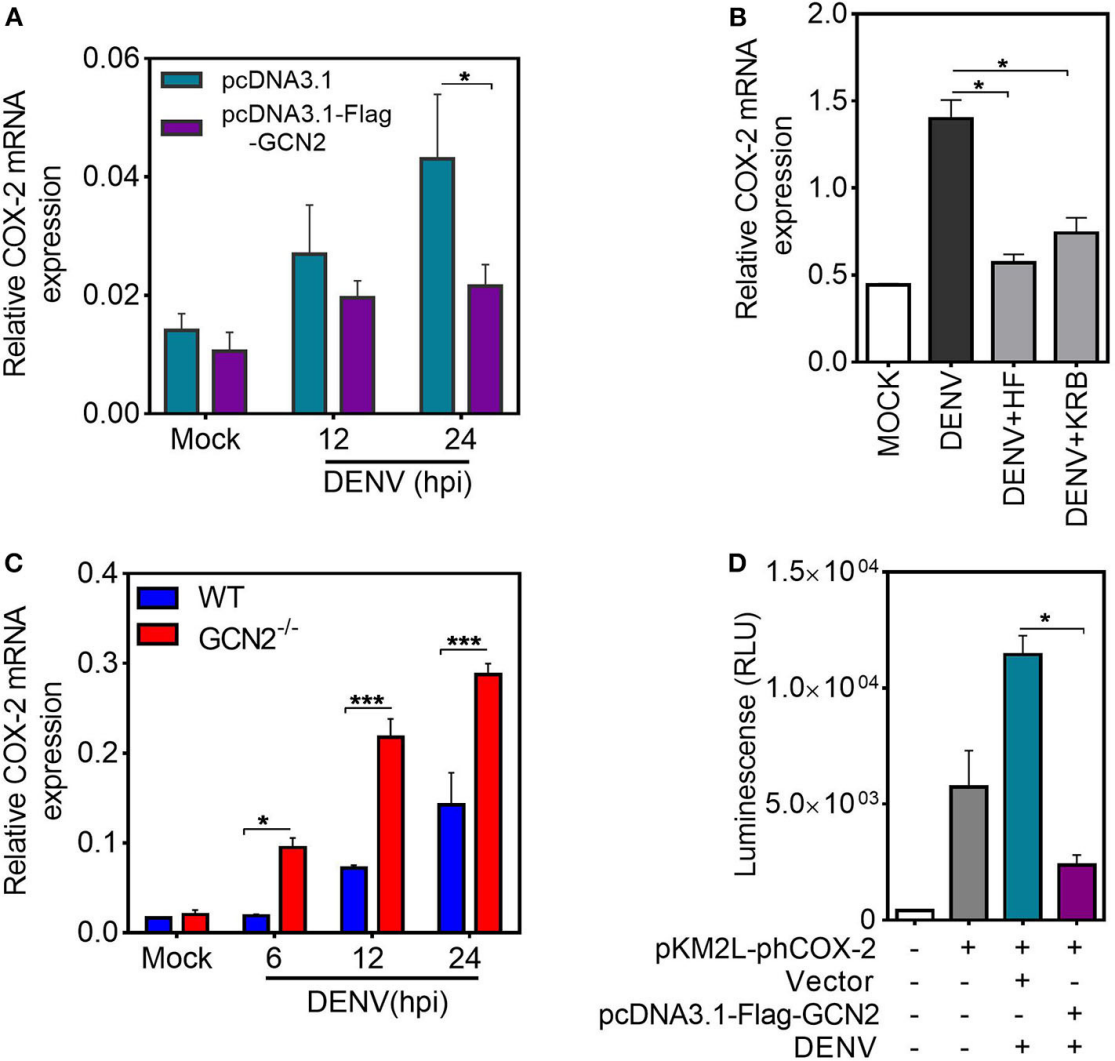
GCN2 inhibits DENV induced COX-2 levels by affecting its transcription. **(A)** HepG2 cells were transfected with pcDNA3.1-Flag-GCN2 plasmid or vector control followed by DENV-2 (moi 3) infection for the indicated time points. COX-2 mRNA expression was checked through qRT-PCR. **(B)** COX-2 mRNA levels in DENV-2 (moi 3) infected HepG2 cells under GCN2 activation conditions using HF (40 nM) or 1X KRB. DENV infection was given for 36 h followed by treatment with HF for 3 h or replacement of growth medium with 1 ml of 1X KRB for 2 h. **(C)** Quantification of COX-2 mRNA in DENV-2 (moi 3) infected WT and GCN2^−/−^ MEFs through qRT-PCR. **(D)** Renilla luminescence levels in HepG2 cells co-transfected with a luciferase reporter plasmid containing human COX-2 promoter (pKM2L-phCOX-2)and pcDNA3.1-Flag-GCN2 plasmid or vector control for 36 h followed by DENV-2 (moi 3) infection for further 24 h. Graphs show data as mean ± SEM from three independent experiments. *P < 0.05, ***P < 0.001 was considered significant. Statistical analysis was done using two-tailed unpaired Student's *t-*test **(A,B,D)** and 2-way ANOVA **(C)**. The COX-2 mRNA expression was normalized to housekeeping gene GAPDH.

It is well-established that COX-2 can be regulated by both transcriptional and post-transcriptional events (28). To examine the mechanism by which GCN2 downregulates COX-2 expression, we first quantified COX-2 mRNA levels upon GCN2 overexpression in HepG2 cells. In accordance with the protein levels, there was substantial reduction in COX-2 mRNA levels in cells overexpressing GCN2 upon DENV-2 infection as compared to vector control (Figure 3A). Similarly, a pronounced reduction in COX-2 mRNA was also observed in DENV-2 infected HepG2 cells under the conditions of GCN2 activation using non-toxic concentrations (Figure S4) (15) of pharmacological activator, Halofuginone (HF) or a well-known GCN2 activator, Krebs Ringer Buffer (KRB) (29) (Figure 3B). Conversely, COX-2 mRNA levels were significantly enhanced in DENV infected GCN2^−/−^ MEFs as compared to WT MEFs upon DENV-2 infection after 6, 12, and 24 h (Figure 3C). These results indicate that GCN2 inhibits COX-2 expression by limiting the expression of its mRNA.

To further investigate whether GCN2 mediated inhibition of COX-2 mRNA is a result of post-transcriptional regulation, HepG2 cells were infected with DENV-2 for 36 h followed by treatment with transcriptional inhibitor Actinomycin-D (Act-D) for 2 h, before addition of HF for 3 h. We observed no significant reduction in COX-2 mRNA transcripts in DENV-2 infected cells upon HF addition as compared to DENV-2 alone in Act-D treated HepG2 cells (Figure S5A) which implies that GCN2 has no role to play in the post-transcriptional regulation of COX-2 mRNA, suggesting that GCN2 may affect COX-2 transcription. To further follow up on this observation, we determined the impact of GCN2 on COX-2 promoter activity. We co-expressed a 1,668 bp segment of the COX-2 promoter cloned in Renilla luciferase reporter vector pKM2L along with GCN2 or vector control followed by DENV-2 infection. GCN2 significantly inhibited COX-2 promoter activity in DENV-2 infected cells as indicated by the prominent reduction in luciferase activity in GCN2 overexpressed cells compared to vector control (Figure 3D). Further, COX-2 promoter activity was also reduced in DENV infected HepG2 cells under conditions of GCN2 activation using HF (Figure S5B). Altogether these results suggest that GCN2 regulates COX-2 expression at the level of transcription and thus affects COX-2 mRNA expression during DENV infection.

### GCN2 Inhibits COX-2 Signaling by Counteracting Activation of the NF-κB Pathway

**Figure 4:**
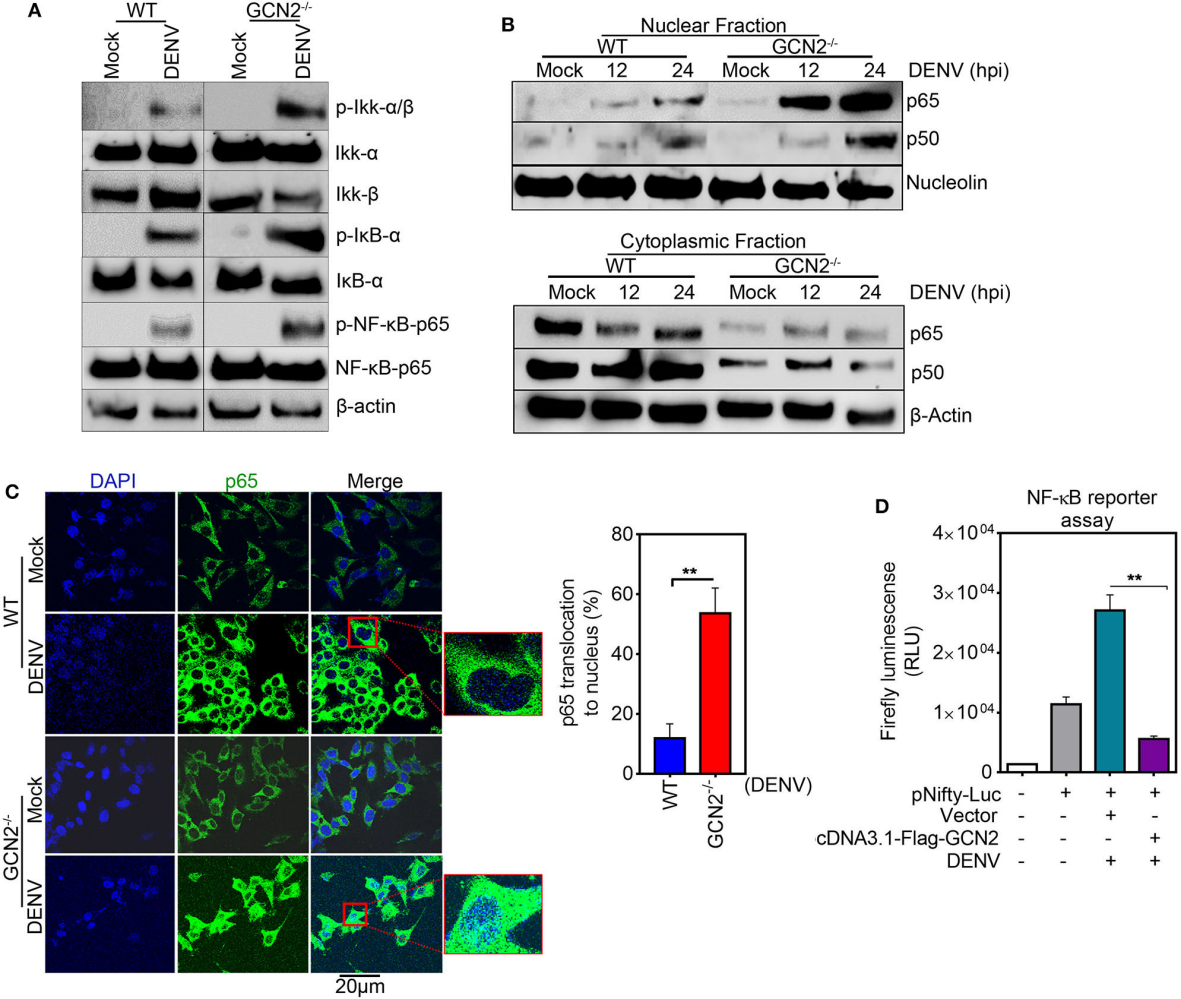
GCN2 interferes with DENV induced COX-2 signaling by counteracting the activation of NF-κB pathway. **(A)** WT and GCN2^−/−^ MEFs were mock infected or infected with DENV-2 (moi 3) for 24 h and examined for the expression of p-IKK-α/β, IKK-α, IKK-β, p-IKB-α, IKB-α, p-NF-κB-p65, and NF-κB-p65 through immunoblotting. **(B)** Immunoblot analysis of p65 and p50 accumulation in nuclear and cytoplasmic fraction of DENV-2 (moi 3) infected WT and GCN2^−/−^ MEFs. **(C)** Confocal microscopy image showing the nuclear translocation of p65 in DENV-2 (moi 3) infected WT and GCN2^−/−^ MEFs 24h post infection. Bar graph represents quantification of percentage of p65 translocation using Image J (NIH) software. Data shown is mean ± SEM from 10 different fields of three independent experiments **(D)** Firefly luminescence levels in HepG2 cells co-transfected with NF-κβ-responsive plasmid (pNifty-Luc) and pcDNA3.1-Flag-GCN2 plasmid or vector control for 36 h followed by DENV-2 (moi 3) infection for further 24 h. Graph represents data as mean ± SEM from two independent experiments performed in triplicates. **P < 0.01 was considered significant. Statistical analysis was done using two-tailed unpaired Student's *t-*test.

To explore the underlying mechanisms of GCN2 mediated control of COX-2 expression, we examined the effect of GCN2 activation on the signaling cascades upstream of COX-2/PGE_2_ activation during DENV infection. It has been shown that NF-κB serves as the central mediator and one of the key transcription factors that regulates COX-2 expression during DENV infection (13, 14). NF-κB forms a central regulator of a number of genes known to influence the host innate immune response and therefore becomes a crucial target of manipulation by pathogens (30). Furthermore, activation of the NF-κB signaling pathway has been shown to be a decisive event favoring COX-2 induced DENV replication and pathogenesis (14). Therefore, we asked whether GCN2 has an impact on NF-κB signaling events upstream of COX-2 synthesis during DENV infection. To unravel the involvement of GCN2 in DENV induced activation of NF-κB signaling, we first examined nuclear translocation of NF-κB subunits p50 and p65 in DENV-2 infected HepG2 cells under GCN2 activation conditions (using different doses of HF). Our results showed a substantial reduction in nuclear p65 and p50 levels in DENV-2 infected cells upon HF treatment (Figure S6A). The activation of NF-κB pathway relies on two upstream phosphorylation events, first being the phosphorylation of Iκβ kinase; IKK-α/β complex; leading to its activation and phosphorylation of the Iκβ inhibitory protein, targeting it for ubiquitination and releasing the NF-κB subunits p50 and p65 (30). Our results showed a reduction in phosphorylation levels of Iκβ kinase IKK-α/β complex, a master regulator of NF-κB activation as well as Iκβ phosphorylation levels in a dose dependent manner upon HF treatment in DENV-2 infected HepG2 cells (Figure S6B). On the contrary, we observed up regulation of the IKK-α/β complex, NF-κB, and Iκβ phosphorylation events in GCN2^−/−^ MEFs as compared to WT-MEFs upon DENV infection (Figure 4A). Concomitantly, immunoblot analysis also showed higher p50 and p65 levels in the nuclear fraction of GCN2^−/−^ MEFs as compared to WT MEFs in a time-dependent manner at 12 and 24 h post DENV-2 infection (Figure 4B). We validated this observation by using immunofluorescence microscopy analysis as well, where we observed GCN2^−/−^ MEFs showing greater accumulation of NF-κB subunit p65 in the nucleus as compared to WT MEFs upon DENV-2 infection (Figure 4C). These results indicate that GCN2 blocks NF-κB activation during DENV infection by suppressing the activity of kinase IKK-α/β complex, thereby inhibiting proteosomal degradation of its substrate Iκβ. To further evaluate the ability of GCN2 to counteract DENV induced NF-κB pathway we assessed NF-κB activity using a luciferase reporter assay, where we co-transfected luciferase reporter plasmid carrying NF-κB responsive elements, along with GCN2 overexpressing construct or respective vector control, followed by DENV-2 infection. Under these experimental conditions, GCN2 significantly inhibited NF-κB activity as observed by the reduction in luminescence levels in GCN2 overexpressed cells as compared to vector control (Figure 4D). Similar results were obtained in DENV-infected HepG2 cells under GCN2 activation conditions using HF. We observed dose dependent reduction in NF-κB luciferase activity in DENV-2 infected cells upon treatment with different doses of HF as compared to only DENV-2 infected cells or those treated with MAZ1310, an inactive derivative of HF (15) (Figure S6C). Also, GCN2 overexpressed cells treated with a known NF-κB inhibitor pyrrolidine dithiocarbamate (PDTC) showed greater reduction in DENV induced COX-2 promoter activity as observed by higher reduction in Renilla luminescence compared to GCN2 overexpressed cells not treated with PDTC (Figure S6D), indicating a synergistic inhibition of NF-κB by PDTC and GCN2 overexpression. This result suggests that GCN2 indeed acts as a potent inhibitor of NF-κB signaling during DENV infection. Altogether, our results suggest that GCN2 inhibits DENV induced COX-2 signaling by counteracting the activation of NF-κB pathway.

## Discussion

It is well-known that viruses are dependent on host protein synthesis machinery for the translation of its RNA (31, 32). In turn, host innate defense machinery activates translation arrest pathways to contain virus replication. Growing evidence suggests that the innate sensor PKR gets activated during various viral infections and upon exposure to synthetic RNA (Poly I:C) (3). However, recent studies highlight that in addition to PKR mediated antiviral responses, the metabolic sensor GCN2 also shows potent antiviral response in a PKR independent manner. For instance, the anti-viral role of GCN2 against various RNA viruses has been documented, including Human Immunodeficiency Virus (16), Vesicular stomatitis virus, and Sindbis virus (9). Moreover, since its discovery, GCN2 has been shown to have multifaceted functions, having implication in a vast array of diseases, including long term memory formation and immune regulation in mammals (33), infection with intracellular bacteria including *Shigella, Salmonella* (29), *and Listeria* (34). However, the physiological relevance of GCN2 during DENV infection is poorly understood.

DENV infection is often termed as *bone-breaking fever* to describe the symptoms elicited in the DENV patients, attributed to the excessive inflammation that plays a crucial role in the pathogenesis and severity of the disease. It has been shown that the COX-2/PGE_2_ signaling cascade is under stringent regulation during DENV infection, and plays a major role in the activation of inflammation-associated pathogenesis of the disease (14). DENV has also been found to programme dendritic cell (DC) migration to various tissues through the regulation of COX-2 signaling cascade (13) during infection conditions. Furthermore, elevated COX-2/PGE_2_ expression has been shown to favor viral replication in case of DENV (14), influenza virus (35), Porcine sapovirus (PSaV) (36), cytomegalovirus (CMV) (37), and hepatitis C virus (HCV) (38). Although, recent studies have revealed the anti-inflammatory functions of GCN2 (15, 39) and have identified GCN2 as a potential target regulating the fate of pro-inflammatory cytokines including IL-1β, IL-17, IFN-γ, and TGF-β (15, 39, 40), the crosstalk between GCN2 and COX-2 signaling during DENV infection remains unexplored.

In the current study, we have described the antiviral potential of GCN2 against DENV and have demonstrated the GCN2 interplay with DENV virus-induced COX-2/PGE_2_ signaling. Our results reveal that GCN2 deficient cells are more susceptible to DENV infection as compared to WT cells. Further, GCN2 contains DENV infection by interfering with DENV replication as depicted by greater dsRNA, a key intermediate in DENV replication cycle, staining in GCN2^−/−^ MEFs as compared to WT MEFs. We report for the first time that GCN2 plays a critical role in restricting DENV induced COX-2 expression and activity as indicated by increased COX-2 levels and PGE_2_ production in DENV infected GCN2^−/−^ MEFs as compared to WT MEFs. Further mechanistic insights indicate that GCN2 activation significantly inhibits COX-2 transcription as revealed by experiments showing a significant reduction in COX-2 promoter activity under GCN2 activation conditions. To the best of our knowledge, this is the first report of an interplay between GCN2 and COX-2 signaling pathways under any pathological conditions. Multiple lines of evidence suggest that the NF-κB transcription factor functions upstream of COX-2 to regulate its synthesis under various conditions, including DENV infection (13, 41). Here we provide evidence that pharmacological activation of GCN2 using HF impedes the activation of Iκκ-NF-κB pathway during DENV infection. Similar to elevated COX-2 levels in DENV infected GCN2^−/−^ cells, we observed a substantial increase in IKK-α/β, IκB phosphorylation, and translocation of NF-κB subunits p50 and p65 in GCN2^−/−^ MEFs indicating that GCN2 suppresses COX-2/PGE_2_ driven inflammation-related pathogenesis during DENV infection by counteracting NF-κB activation. Additionally, inhibition of COX-2 using Celecoxib profoundly suppressed DENV infection in GCN2^−/−^ MEFs suggesting that increased DENV infection in GCN2 deficient cells is due to the elevated levels of COX-2 production in absence of GCN2.

Next step could be to further study the strategies employed by DENV to counteract the antiviral effect of GCN2. Our preliminary results show that DENV inhibits eIF2α phosphorylation in WT MEFs as the infection progresses which was exacerbated by the absence of GCN2, suggesting that GCN2 regulates this process (Figure S7). GCN2 has previously been shown to set the platform for the activation of downstream host anti-viral responses *via* eIF2α phosphorylation, it would be further interesting to decipher how DENV modulates GCN2- eIF2α axis to evade GCN2 mediated antiviral effect.

**Figure 5:**
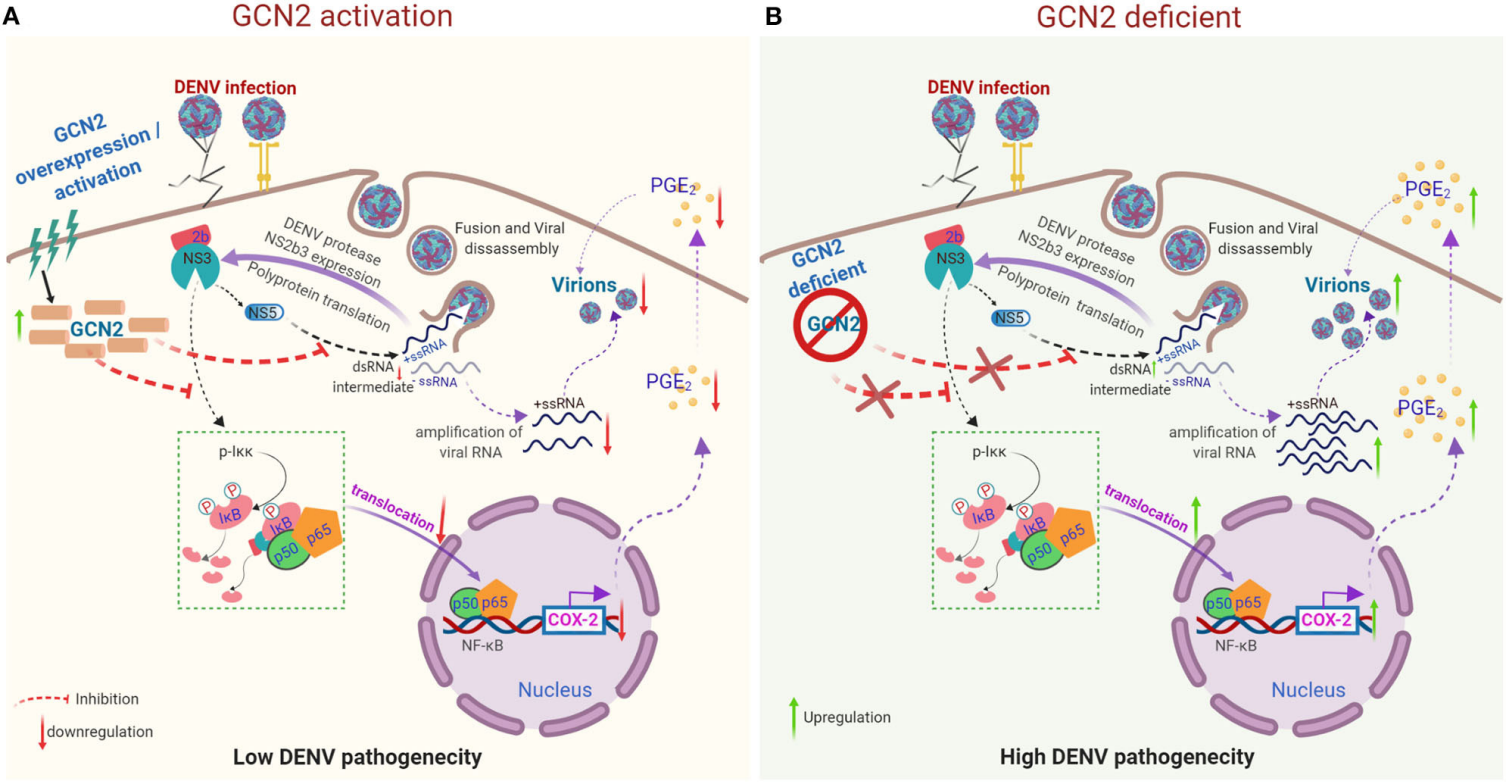
Proposed model representing the mechanism by which GCN2 suppresses DENV induced COX-2/PGE_2_ signaling and its pathogenesis. **(A)** Upon activation, GCN2 suppresses DENV replication and interferes with the nuclear translocation of NF-κB subunits thereby suppressing COX-2/PGE_2_ production and COX-2 mediated DENV pathogenesis. Additionally, GCN2 abrogates DENV replication by interfering with the production of its dsRNA intermediate. **(B)** Under GCN2 deficient condition, DENV induces enhanced expression of COX-2 and increased PGE_2_ production. Further, there is increased DENV RNA amplification and mature virion production resulting in enhanced DENV pathogenesis.

In conclusion, our results describe a molecular mechanism by which activation of GCN2 pathway can interfere with COX-2/PGE_2_ mediated inflammation and DENV pathogenesis (Figure 5). These results for the first time provide evidence supporting an anti-viral effect of GCN2 against a virus belonging to *Flavivirus* family (Figure 5). Altogether, our data points toward a novel and physiologically significant role for GCN2 in innate control of DENV infection and progression.

## Data Availability Statement

All datasets presented in this study are included in the article/Supplementary Material.

## Author Contributions

NK conceptualized the study. SA propagated all serotypes of DENV, quantified them, and performed most of the experiments with help from SB. NK, SA, and SB wrote the manuscript, analyzed, and interpreted the data. JG helped in making figures. All authors read and approved the manuscript for submission.

## Conflict of Interest

The authors declare that the research was conducted in the absence of any commercial or financial relationships that could be construed as a potential conflict of interest.
