# Supplementary material for: Dengue Virus Induced COX-2 Signaling Is Regulated Through Nutrient Sensor GCN2
Source: Front Immunol. 2020 Aug 13;11:1831. doi: 10.3389/fimmu.2020.01831 (PMC7438581; doi:10.3389/fimmu.2020.01831)
Supplement: Supplementary file 1 [file Data_Sheet_1.pdf]

Supplementary Information

Dengue virus induced COX-2 signaling is regulated through nutrient sensor GCN2

Sumbul Afroz[1], Srikanth Battu[1, 2], Jeevan Giddaluru[1] and Nooruddin Khan[1, 3*]

[1]Department of Biotechnology and Bioinformatics, School of Life-Sciences, University of Hyderabad, Hyderabad-500046, Telangana, India.

[2]Laboratory of Molecular Cell Biology, Centre for DNA Fingerprinting and Diagnostics (CDFD), Inner Ring Road, Uppal, Hyderabad-500039, Telangana, India.

[3]Department of Animal Biology, School of Life-Sciences, University of Hyderabad, Hyderabad-500046, Telangana, India.

[*]Correspondence should be addressed to: Nooruddin Khan

Dr. Nooruddin Khan
Associate Professor,
Department of Animal Biology,
University of Hyderabad.
Hyderabad-500046, Telangana, India.
(noor@uohyd.ac.in).

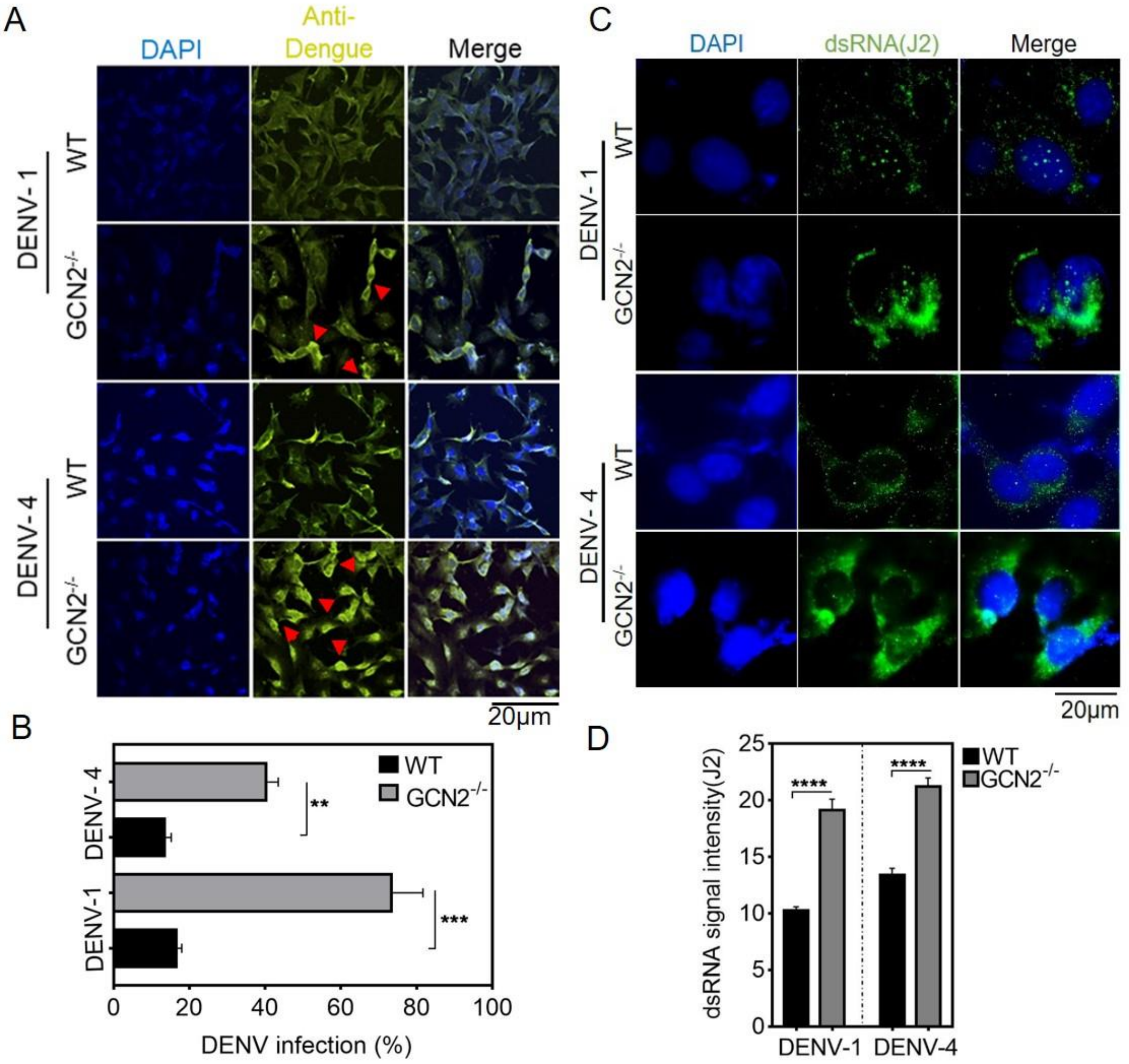

Figure S1. GCN2 deficient cells are highly susceptible to infection with other DENV serotypes. (A-B) WT and GCN2$^{-/-}$ MEFs were mock infected or infected with DENV-1 and DENV-4 (moi 3) for 36h for analysis of DENV pathogenesis. The cells were immunostained with Anti-Dengue (green) antibody to stain infected cells. (A) Confocal microscopy image showing the number of DENV-1 and DENV-4 positive cells. (B) Quantification of percentage of DENV-1 and DENV-4 infection in WT and GCN2$^{-/-}$ MEFs in ten different fields using Image J (NIH) software. **(C)** Analysis of dsRNA accumulation in DENV-1 and DENV-4 (moi 3) infected WT and GCN2$^{-/-}$ MEFs at 36hpi by immunofluorescence. J2 antibody was used to detect DENV dsRNA intermediate. Alexa-Fluor 488 conjugated secondary antibody was used to detect primary antibody respectively. **(D)** Quantification of dsRNA (J2 signal intensity) using Image J software. Data is mean ± SEM of three independent set of experiments. *P< 0.05, **P< 0.0, ***P<0.001, ****P<0.0001 was considered significant. Statistical analysis was done using two-tailed unpaired Student's *t* test.

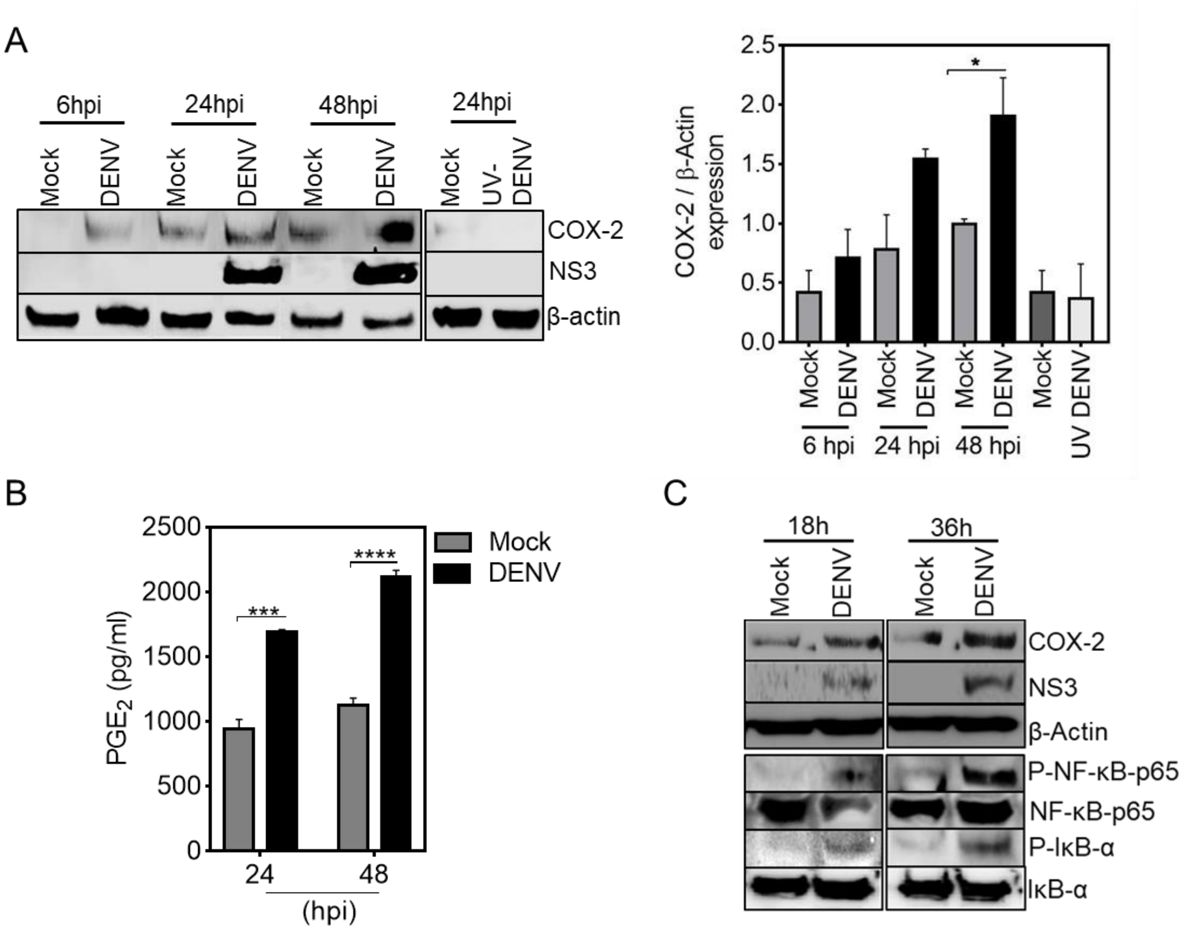

Figure S2. DENV enhances COX-2 expression. (A-B) HepG2 cells were mock infected or DENV-2 (moi 3) infected or infected with equal moi of UV treated DENV-2. Cell lysates were prepared or supernatants were collected at indicated time points. (A) Immunoblot analysis of COX-2 expression in cell lysates (left). Densitometry analysis of immunoblot showing COX-2 expression levels. The expression of COX-2 protein was normalized to β-actin using Image J software (right). Graph is representative of mean ± SEM of three independent experiments (B) PGE_2 level was estimated in culture supernatants by ELISA. Graph represents data as mean ± SEM from two independent experiments performed in triplicates. *P<0.05, ***P<0.001, ****P<0.0001 was considered as statistically significant. Statistical significance was calculated by two-tailed unpaired Student's t-test (A) and 2-way ANOVA (B). **(C)** Primary human monocytes were mock infected or infected with DENV-2 at moi=3 and expression of COX-2, P-NF-κβ-p65, NF-κβ-p65, P-Iκβ-α and Iκβ-α was checked at the indicated time points. Immunoblot analysis of the expression levels of above mentioned proteins in cell lysates. β-actin was used as loading control.

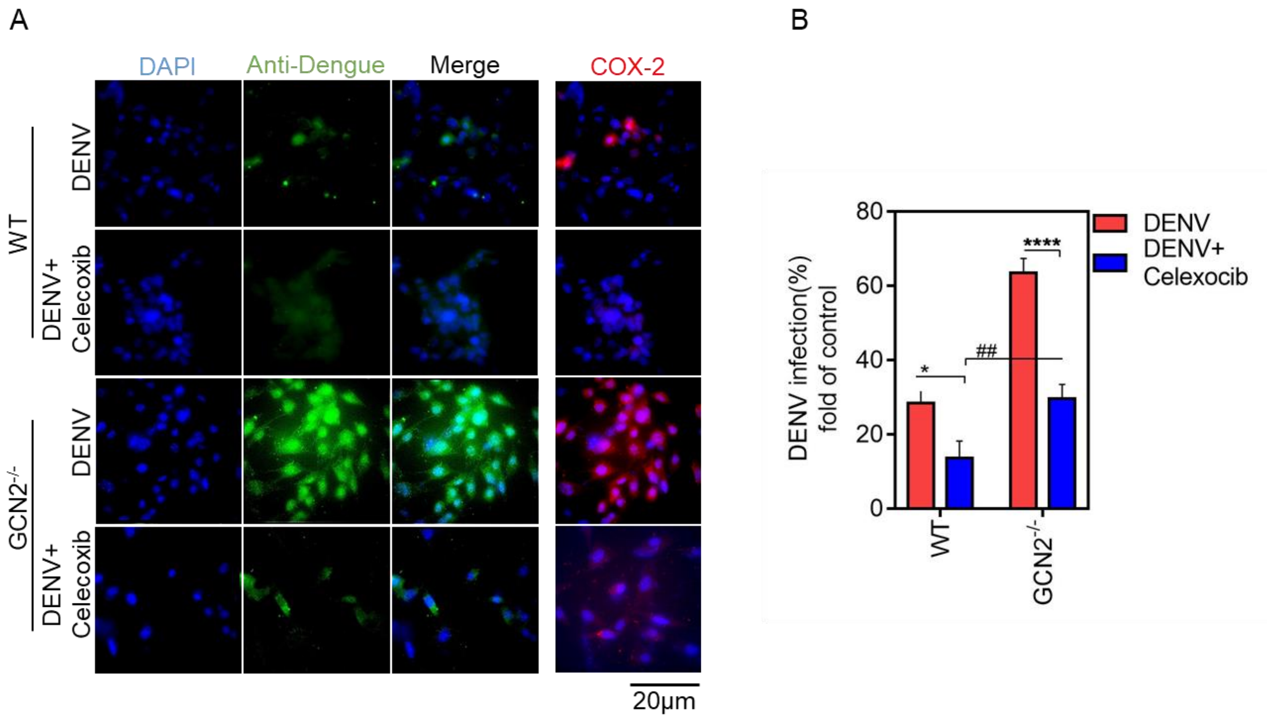

Figure S3. GCN2 shows anti-viral activity against DENV by limiting COX-2 production. (A) WT and GCN2$^{-/-}$ MEFs were mock infected or infected with DENV-2 (moi 5) for 36h with or without COX-2 inhibitor Celecoxib (10µM) for analysis of DENV pathogenesis. Immunofluorescence image is representative of one of 3 independent experiments. **(B)** Quantification of no. of DENV-2 infected cells using Image J software. Data is mean ± SEM of three independent experiments.*P< 0.05, ***P<0.001, ## P<0.01 was considered significant. Statistical analysis was done using two-tailed unpaired Student's *t* test.

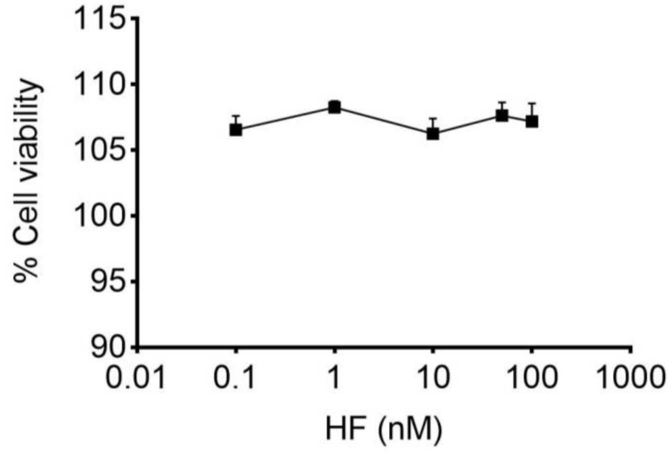

Figure S4. HF is not toxic to HepG2 cells at concentrations up to 100nM. HepG2 cells were treated with different concentrations of HF for 48h. Cell cytotoxicity of HF was assessed by MTT assay. Data is from two independent experiments.

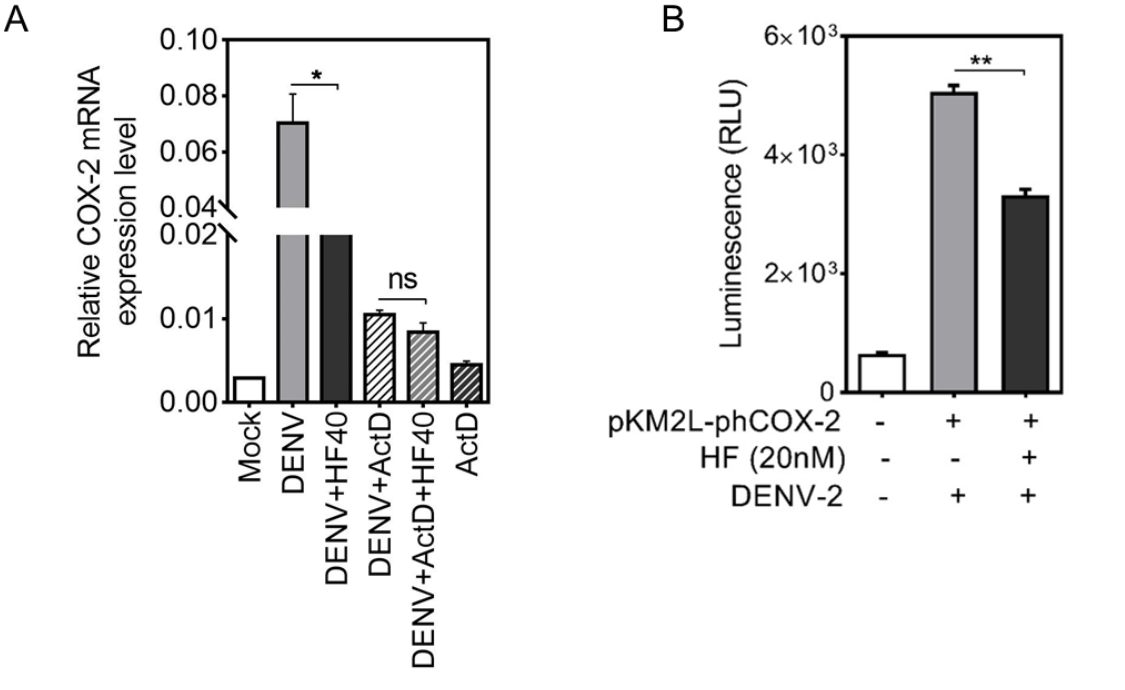

Figure S5. GCN2 regulates DENV induced COX-2 mRNA expression at transcriptional level. (A) HepG2 cells were infected with DENV-2 (moi 3) for 36h followed by Actinomycin D (ActD, 10µg/ml) treatment for 2h. Post ActD treatment, HF (40nM) was given for 3h. COX-2 mRNA levels was quantified through qRT-PCR. Graph represents data as mean ± SEM from three independent experiments. *P< 0.05 was considered significant. Statistical significance was calculated by two-tailed unpaired Student's t-test. **(B)** Renilla luminescence levels in HepG2 cells transfected with a luciferase reporter plasmid containing human COX-2

promoter (pKM2L-phCOX-2) for 36h followed by DENV-2 (moi 3) for further 24h. HF treatment was given in the last three hours of infection. Graph represents data as mean ± SEM from three independent experiments. **P<0.01 was considered significant. Statistical significance was calculated by two-tailed unpaired Student's t-test.

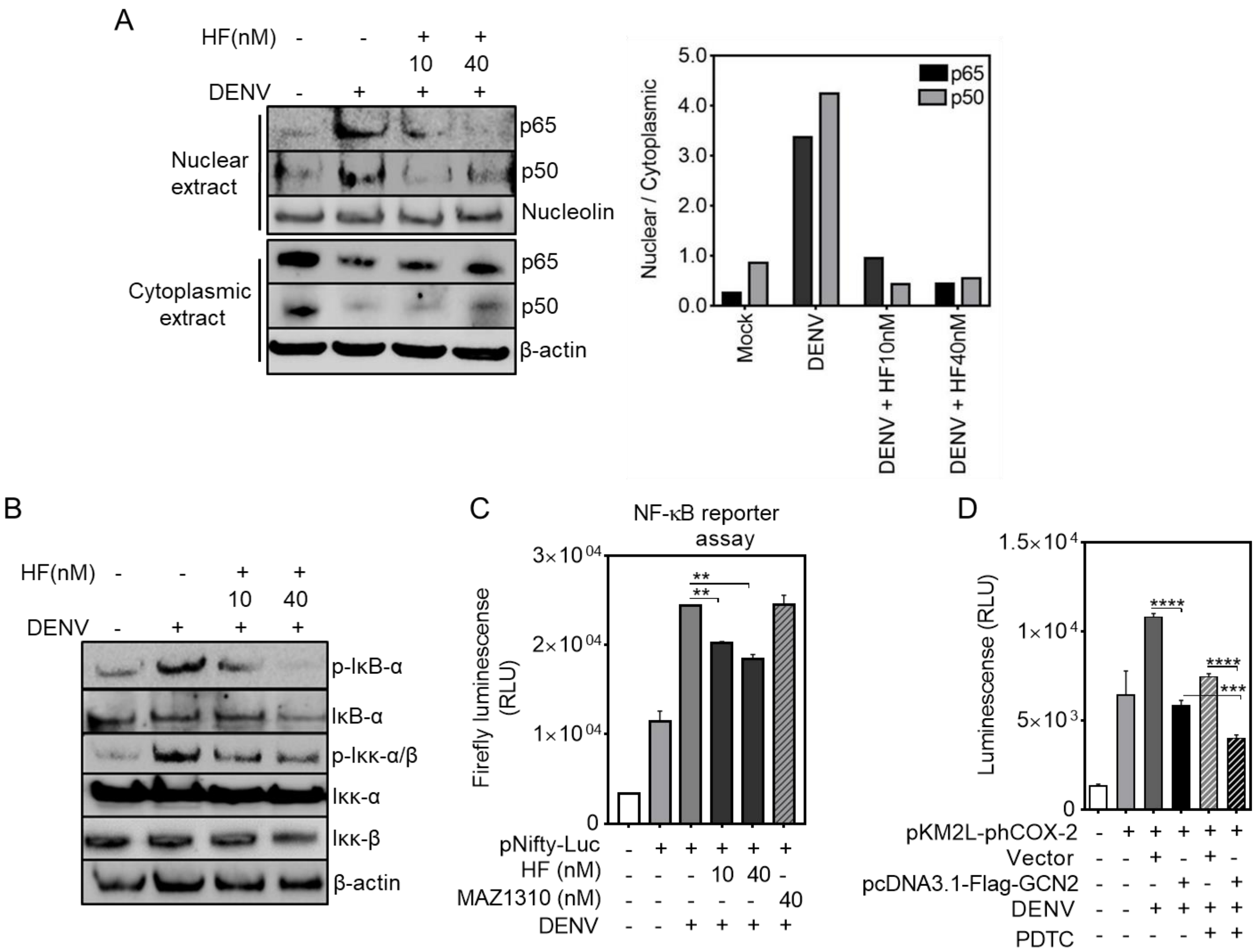

Figure S6. DENV induced NF-κβ signaling is dampened under GCN2 activation conditions. (A) Immunoblot and densitometric analysis of p65 and p50 accumulation in nuclear and cytoplasmic fraction of HepG2 cells infected with DENV-2 (moi 3) followed by treatment with different doses of HF. Blot represents data from one out of N=3 independent experiments. **(B)** Immunoblot analysis of p-IKB-α, IKB-α, p-IKK-α/β, IKK-α, IKK-β expression in HepG2 cells infected with DENV-2 (moi 3) followed by treatment with different doses of HF. Blot represents data from one out of three independent experiments. **(C)** Firefly luminescence levels in HepG2 cells transfected with NF-κβ responsive plasmid

(pNifty-Luc) for 36h followed by DENV-2 (moi 3) infection for further 24h. HF or its inactive derivative MAZ1310 treatment was given in the last three hours of infection. **(D)** Renilla luminescence levels in HepG2 cells co-transfected with a luciferase reporter plasmid containing human COX-2 promoter (pKM2L-phCOX-2) and pcDNA3.1-Flag-GCN2 plasmid or vector control for 36h. The transfected cells were further treated with or without PDTC (50µM) for 2h prior to DENV-2 (moi 3) infection for further 24h. PDTC (50µM) treatment was maintained for the infection duration. Graphs in (C) and (D) represent data as mean ± SEM from two independent experiments, each with three replicates. **P<0.01, ***P<0.001, ****P<0.001 was considered significant. Statistical analysis was done using two-tailed unpaired Student's *t* test.

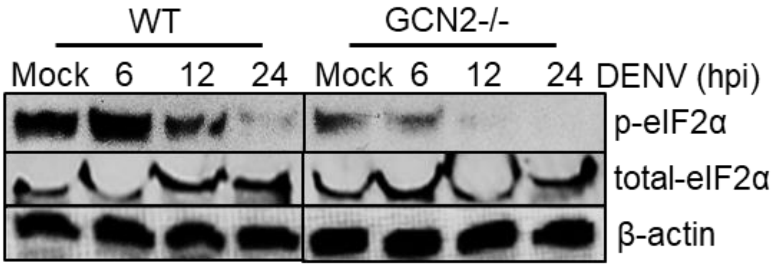

Figure S7. DENV inhibits eIF2α phosphorylation. WT or GCN2$^{-/-}$ MEFs were mock infected or infected with DENV-2 at moi=3 and lysates were prepared at 6, 12 and 24 hours post infection (hpi). Immunoblot analysis of eIF2-α phosphorylation in the cell lysates.β-actin was used as loading control.
